# Nanostructure Engineering by Oblique Angle Deposition for Photodetectors and Other Applications

**DOI:** 10.3390/mi16080865

**Published:** 2025-07-27

**Authors:** Gyeongho Lee, Raksan Ko, Seungme Kang, Yeong Jae Kim, Young-Joon Kim, Hocheon Yoo

**Affiliations:** 1 Semiconductor Total Solution Center, Korea Institute of Ceramic Engineering and Technology, 3321 Gyeongchung-daero, Icheon 17303, Republic of Korea; 2 Department of Materials Science and Engineering, Korea University, 145 Anam-ro, Seoul 02841, Republic of Korea; 3 Department of Semiconductor Engineering, Gachon University, 1342 Seongnam-daero, Seongnam 13120, Republic of Korea; 4 Department of Electronic Engineering, Gachon University, 1342 Seongnam-daero, Seongnam 13120, Republic of Korea; 5 Department of Electronic Engineering, Hanyang University, 222 Wangsimni-ro, Seoul 04763, Republic of Korea

**Keywords:** oblique angle deposition, nanostructure, photodetectors

## Abstract

Oblique angle deposition (OAD) holds significant potential for diverse applications, including energy harvesting devices, optoelectronic sensors, and electronic devices, owing to the creation of unique nanostructures. These nanostructures are characterized by their porosity and nanoscale columns, which can exist in numerous forms depending on deposition conditions. As a result, the engineering of nanostructures using OAD achieves the successful modulation of optical properties such as absorption, reflection, and transmission. This explains the current surge of attention toward photodetectors based on OAD technology. This review presents various photodetectors based on OAD technology and summarizes reported cases. It also explores current advancements, major applications, and future directions in photodetector development and nanostructure engineering. Ultimately, this review aims to provide a comprehensive overview of the research trends in photodetectors utilizing OAD technology and focus on their further development and application potential.

## 1. Introduction

Nanostructure engineering is attracting a lot of attention because of its potential for a wide range of useful applications [1,2,3,4]. Various strategies have been explored to achieve functional nanostructures, including planar configurations for controlled lateral growth [5], vertically aligned nanowires with directional charge transport [6], and hetero-integrated one-dimensional (1D) systems that avoid lattice mismatch constraints [7]. While these approaches offer distinct advantages in terms of structural and optoelectronic control, they often require complex epitaxial growth procedures or lithographic patterning. In contrast to traditional heterostructures that demand strict lattice matching to avoid defect formation and strain accumulation, 1D nanostructures fabricated via oblique angle deposition (OAD) can be seamlessly integrated with a variety of substrates without such limitations [7]. This inherent tolerance to lattice mismatch simplifies the fabrication process and enhances device performance by reducing interfacial defects and improving carrier mobility. In this context, OAD offers a particularly attractive fabrication route: it enables lithography-free, substrate-independent, and angle-controlled bottom-up growth of anisotropic nanostructures, making it versatile for scalable integration across diverse optoelectronic platforms.

In particular, OAD, which can build nanostructures in a simple way, is a deposition method that is typically carried out using physical vapor deposition (PVD) [8,9,10,11]. The porous nanocolumns are the representative structures that can be formed by OAD, and different shapes of nanostructures can be generated by the deposition conditions of OAD, such as zigzag [12,13], helical [14,15], and vertically aligned [16,17,18] structures. In particular, the ability to control the optical properties (absorption, reflection, and transmission) and surface morphology is the main benefit of OAD films through these structures [19,20,21,22]. Therefore, OAD technique is suitable for widespread use in light-based devices, followed by bio/gas sensing [23,24,25,26], solar cells [27,28,29,30], and water splitting [31,32,33] applications.

Meanwhile, a photodetector is a device that converts optical signals into electrical signals by absorbing light and producing a change in current. The materials used for an active layer and the operating principle determine the types of photodetectors, such as metal–semiconductor–metal (MSM) photodetector [34,35,36], photodiode [37,38,39], and phototransistor [40,41,42]. In OAD-based photodetectors, two-terminal structures are typically used. OAD technique either forms the active layer or enhances light absorption, thereby improving photoresponse through nanostructure engineering [43,44,45].

Here, we focus on the optical properties of reported photodetectors utilizing OAD technology and distinguish them according to different deposition methods. Accordingly, the three sections provided are as follows: (i) basic OAD design and optical property modulation mechanism; (ii) OAD photodetector; and (iii) applications of OAD photodetector. Consequently, our aim is to review the enhanced performance of photodetectors achieved through the OAD method reported to date and to provide a comprehensive discussion on the actively researched application areas and the future directions of OAD technology.

This review article offers a fabrication- and morphology-oriented new perspective on photodetectors. We examine how OAD enables the engineering of nanostructures such as nanorods, nanopillars, and porous films and how these morphologies influence optical and photoresponsivity performance. Furthermore, by systematically categorizing OAD-based photodetectors according to the deposition method (e.g., e-beam, thermal, sputtering, and pulsed laser deposition (PLD)), this review provides insight into the relationship between fabrication technique, structure, and function. We also expand the discussion beyond photodetectors to include emerging applications of OAD-engineered nanostructures in solar cells, anti-reflective coatings, and neuromorphic devices. Specifically, the outline of this article is as follows: Section 1 introduces the fundamentals of OAD and highlights its significance in photodetector applications. Section 2 presents the basic principles of OAD design and the underlying mechanisms of optical property modulation. Section 3 categorizes OAD-based photodetectors according to four major PVD methods and examines their performance characteristics. Section 4 explores extended applications of OAD-engineered nanostructures, including anti-reflective coatings, solar cells, and neuromorphic devices. Section 5 summarizes the key insights from this review and outlines future directions for OAD-enabled device development.

## 2. Basic OAD Design and Optical Property Modulation Mechanism

OAD includes all techniques in which the deposition flux is directed at a specific angle to the substrate as the target material evaporates and reaches the substrate, also known as glancing angle deposition (GLAD) [46,47] or ballistic deposition [48,49]. As mentioned in the Introduction, the different types of nanostructures could be formed due to the shadowing effect, most commonly with porous nanocolumn (NC) structures including nanorods (NRs) and nanowires (NWs) [50,51,52,53,54].

**Figure 1:**
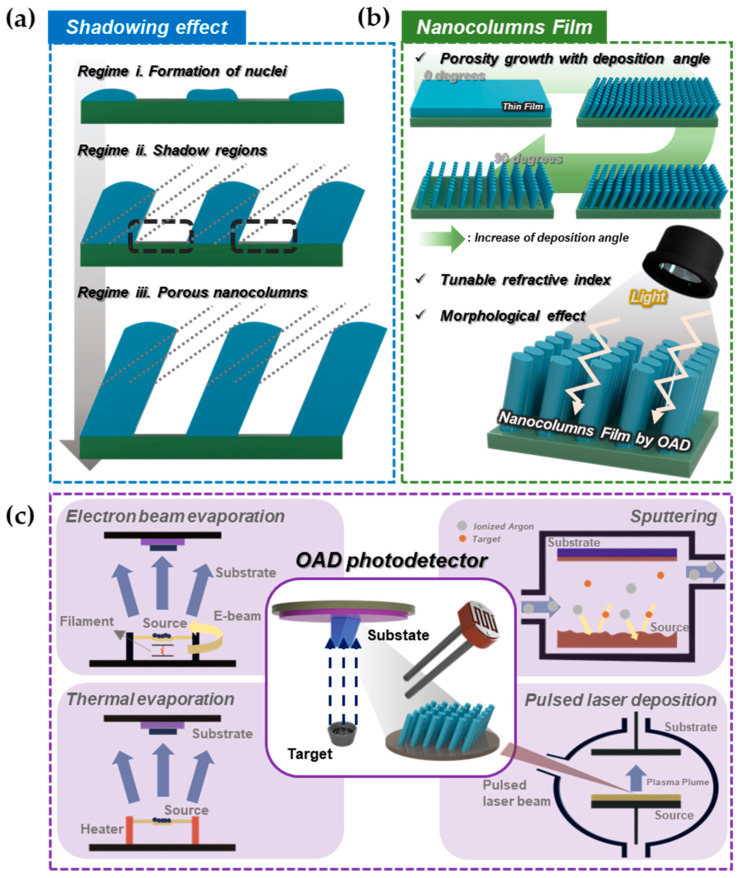
(**a**) Mechanistic illustration for shadowing effect. Each regime describes formation of nuclei, shadow regions, and porous nanocolumns, respectively. (**b**) Porosity growth with increasing deposition angle, and properties of porous nanocolumns film. (**c**) Schematic illustration of OAD photodetector depending on deposition methods (e-beam evaporation, thermal evaporation, sputtering, and pulsed laser deposition).

As shown in Figure 1a, the formation process of these nanostructures by the shadowing effect is divided into three regimes as follows: (i) the beginning of the shadowing effect is the formation of nuclei; (ii) as the nuclei grow, the shadowed regions between the nuclei are generated; and (iii) specially, because the target material is positioned at a certain angle with the substrate, the nuclei, which continue to grow, form columns and the regions in the shadows remain empty spaces. Consequently, the porous nanocolumns film is obtained by the shadowing effect [55,56,57,58]. Furthermore, there are several types of parameters involved in the formation of nanostructures in OAD technique, including deposition rate, substrate species, target materials, and rotation speed [59,60,61]. Within these parameters, the deposition angle has the most significant impact on the unique nanostructure of the OAD film, revealing useful features such as porosity, birefringence, and magnetic anisotropy [62,63,64]. These features can be used to modulate the optical properties by controlling the optical coefficients. Specifically, reflectance can be expressed mathematically by refractive index according to Fresnel’s equation when incident light passes through different media [65,66]. Generally, the refractive index of air, which has a value of 1.0003, is lower than that of semiconductors [67,68]. In the case of OAD film, there are lots of spaces that are occupied by air due to the porosity, and then the refractive index of the entire OAD film becomes lower than that of the thin film (TF). Thus, the reflectance of OAD film, which has a porous NC structure, can be modified by modulating the porosity of the film through the deposition angle [69]. Moreover, the morphology of the nanostructures affects how light interacts with the film. The degree of reflection, scattering, absorption, and transmission can change. Consequently, light absorption in an OAD photodetector can be enhanced by modulating optical properties via porosity and morphology engineering. Figure 1b exhibits the changing porosity of OAD film with increasing deposition angle and an NC film describing the properties of a nanocolumn film by OAD (tunable refractive index and morphological effect).

To produce various types of nanostructured films, PVD process is mainly used because the particle flow is highly directional and it is easy to control the incident deposits at specific angles, and this method can effectively control the shadowing effect to form porous NC films [8]. Thus, OAD technology has been actively studied using PVD technology for the past several decades, and many attempts have been made to fabricate nanostructures using electron beam (e-beam) evaporation [70], thermal evaporation [71], sputtering [72,73], and PLD [74,75]. In this review, we report OAD photodetectors developed by four types of PVD methods (e-beam evaporation, thermal evaporation, sputtering, and PLD) (Figure 1c). These nanostructures can be precisely engineered by tuning deposition angle, rotation, and substrate motion, offering porosity control, alignment, and spacing.

## 3. OAD Photodetector

To offer a fabrication-based perspective, this review classifies OAD-based photodetectors by the deposition method, discussing how each technique affects the resulting nanostructure and device performance.

### 3.1. Performance Parameters of Photodetectors

In general, there are parameters (sensitivity, responsivity, external quantum efficiency (EQE), detectivity, rise time, and decay time) that can be used to assess the performance of photodetectors [76,77,78,79]. Sensitivity is a value that measures how sensitive the photodetector is to the wavelength of the target light. Sensitivity is calculated from the equation as follows:

(1) $$S\;=\;\frac{I_{ph}-\;I_{dark}}{I_{dark}}\;(A{\cdot A}^{-1})$$

where $I_{dark}$ is the dark current, while $I_{ph}$ is the photocurrent under light irradiation.

The sensitivity in a photodetector is often expressed as the photo-to-dark current ratio (*PDCR*). *PDCR* simply represents the ratio of the photocurrent to the dark current and calculated from the equation as follows:

(2) $$PDCR=\;\frac{I_{ph}}{I_{dark}}\;\left(A{\cdot A}^{-1}\right)$$

where $I_{dark\;}$ and $I_{ph}$ have the same meaning as mentioned above, and based on the above formula, sensitivity is equal to the value obtained by subtracting 1 from *PDCR*.

Responsivity is the main parameter for evaluating the performance of a photodetector and represents its photoresponse to light. Responsivity is calculated from the equation as follows:

(3) $$R\;=\;\frac{I_{ph}-\;I_{dark}}{P_{in}\cdot S}\;(A{\cdot W}^{-1}),$$

where S refers to the incident area of light, and $P_{in}$ denotes the power of incident light.

EQE is the number of electrons produced per photon incident on the photo detector. EQE is calculated from the following equation:

(4) $$\eta_{e}\;=\;R\frac{h\cdot c}{e\cdot \lambda }\;\times 100\;(\%),$$

where h is Planck’s constant, c is speed of light, *e* is electron charge, λ is wavelength of incident light, and R is responsivity of the photodetector.

Detectivity depends on responsivity and noise equivalent power (NEP), which can be calculated according to the following equation:

(5) $$D^{*}\;=\;\frac{R}{\sqrt{2eJ_{dark}}}\;(Jones),$$

where $J_{dark}$ is the dark current density.

Finally, rise time refers to the time when the current change by photoresponse reaches 10% to 90%, while decay time indicates the time when the current change reaches 90% to 10% after light illumination.

### 3.2. OAD Photodetectors Deposited by E-Beam Evaporation

**Figure 2:**
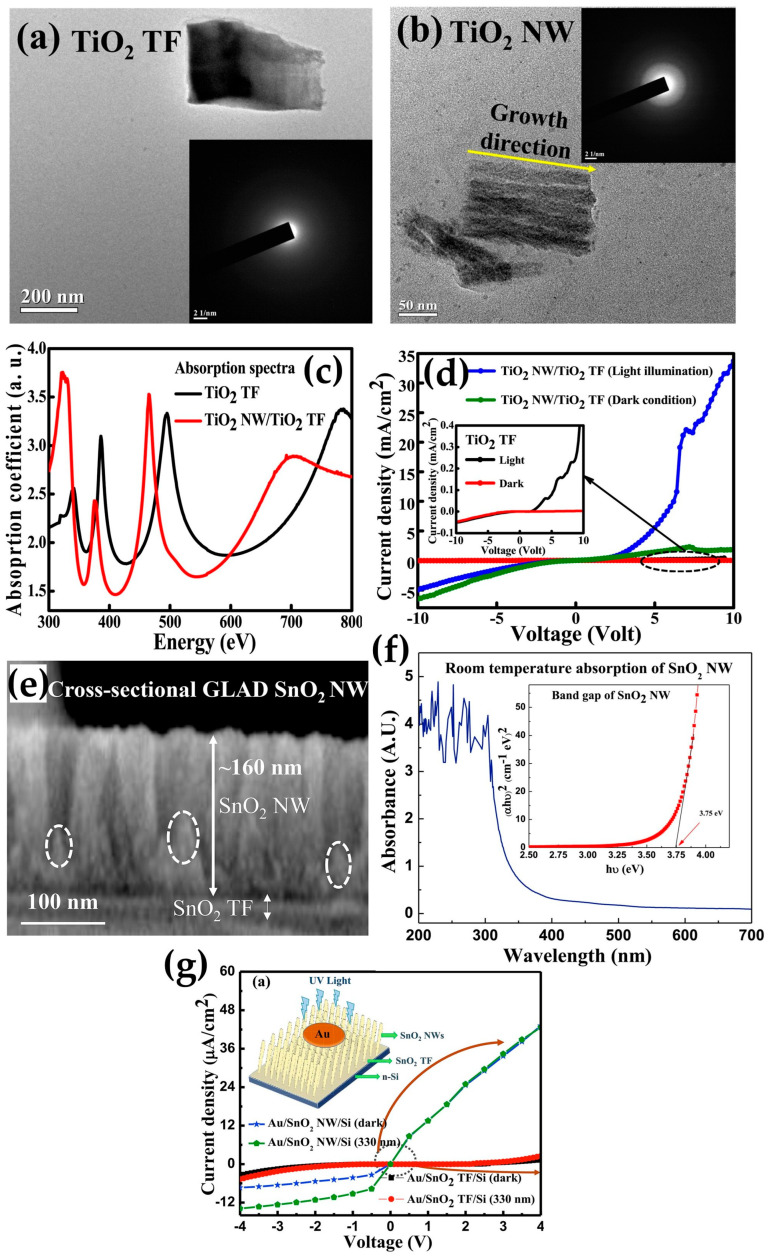
TEM images of (**a**) TiO_2_ TF and (**b**) TiO_2_ NW/TiO_2_ TF. (**c**) Absorption spectra of TiO_2_ TF and TiO_2_ NW/TiO_2_ TF devices measured by UV-Vis spectrometry. (**d**) J-V curves of TiO_2_ NW/TiO_2_ TF device in dark and light conditions. Inset shows J-V curves of TiO_2_ TF device in dark and light conditions, which has almost no photoresponse compared to TiO_2_ NW/TiO_2_ TF device (adapted from [83] with permission from Elsevier B.V.). (**e**) Cross-sectional SEM image of SnO_2_ NW formed by OAD, showing formation of distinct nanostructure by OAD. (**f**) Absorption spectrum of SnO_2_ NW film. Inset shows band gap for SnO_2_ NW. (**g**) J-V curves of SnO_2_ NW and SnO_2_ TF devices, showing extent of photoresponse for each device (adapted from [84] with permission from Elsevier B.V.).

Electron beam evaporation is a type of PVD in which an electron beam produced by thermionic emission evaporates a target material to form a film [80]. As we mentioned above, the shadowing effect can be effectively controlled by using electron beam evaporation [81,82]. For this reason, Mazumder et al. fabricated a vertically aligned TiO_2_ NW photodetector by electron beam-assisted OAD and compared it to a TiO_2_ thin film (TF) device [83]. As shown in the TEM image in Figure 2b, the vertically aligned TiO_2_ NW sample was formed through deposition at an angle of 85°. Next, the absorption spectrum measured by ultraviolet–visible (UV-Vis) spectroscopy shows larger absorption of the TiO_2_ NW/TiO_2_ TF device than the TiO_2_ TF device under a wavelength of 350 nm (Figure 2c). In addition, the band gap of the TiO_2_ NW/TiO_2_ TF and TiO_2_ TF devices was found to be 3.5 eV and 3.59 eV, respectively, indicating that the values are almost identical regardless of deposition angle. Finally, to understand the impact of OAD on photoresponse of the photodetector, the current density–voltage (J-V) characteristics were assessed under both light (300-W Xenon arc lamp) and dark conditions (Figure 2d). In the dark, the TiO_2_ NW/TiO_2_ TF device indicated the obvious non-linearity behavior, the turn-on voltage of which was 2.4 V, while the non-linearity behavior was not shown in the case of the TiO_2_ TF device. On the other hand, under light conditions, the NW and TF devices turned on at 1.8 V and 1.4 V, respectively, and the difference in the current level between two devices was one hundred times. Consequently, TiO_2_ NW and Au junction generated the good Schottky contact, and it led to the enhanced photoresponse of the TiO_2_ NW/TiO_2_ TF photodetector formed by OAD.

Metal oxide semiconductors with oxygen vacancies are commonly used in photodetectors because the oxygen adsorption and desorption processes help the photoreaction to occur efficiently. Chetri et al. fabricated the self-powered UV photodetector using porous SnO_2_ NW [84]. The SnO_2_ NW was formed by OAD at a deposition angle of 85°, and Figure 2e shows the cross-sectional SEM image depicting the porous NWs. Moreover, the UV-Vis spectroscopy was performed to investigate the absorption region of SnO_2_ NW (Figure 2f). The SnO_2_ NW film shows the absorption in the UV region (200 nm–350 nm), with a band gap of 3.57 eV. To evaluate the performance of self-powered SnO_2_ NW UV photodetector, the J-V characteristics were examined by comparing the SnO_2_ NW device with the SnO_2_ TF device under dark and illuminated conditions (330 nm, 69 μW·cm^−2^) (Figure 2g). In both devices, the non-linearity behavior, representing the rectification properties, was indicated.

However, the larger photocurrent was generated in the case of the SnO_2_ NW device due to the morphology of SnO_2_ NW, which causes light harvesting. Specifically, the generous surface area of SnO_2_ NW, providing the reactive sites with oxygen, facilitates photogenerated carriers (holes) to absorb oxygen. This reaction between SnO_2_ NWs and oxygen molecules results in the interruption of electron–hole pair recombination and improved photoresponse. Notably, the photoresponse was observed at 0 V in the SnO_2_ NW device, suggesting the potential for a self-powered photodetector (Figure 2g). Finally, performance parameters were calculated to evaluate the SnO_2_ NW photodetector. In particular, the sensitivity, responsivity, detectivity, rise time, and decay time at a wavelength of 310 nm were found to be 6.018%, 0.36 mA·W^−1^, 3.02 × 10^9^ Jones, 0.72 s, and 1.79 s to demonstrate the performance of SnO_2_ NW device as a UV photodetector. These results confirm that OAD via e-beam evaporation enables high UV absorption and self-powered operation through enhanced surface area and morphology. However, most of the devices lack detailed electrical stability analysis, and durability and noise characteristics seem to be required for future practical applications.

### 3.3. OAD Photodetectors Deposited by Thermal Evaporation

With the energy of deposition particles kept below 0.3 eV, the thermal evaporation technique allows for the formation of microstructures or crystalline structures in thin films, minimizing the influence of deposition particles [85,86,87]. Additionally, the shadowing effect facilitates effective control over the geometric structures of nano-features, making it a valuable tool for manipulating the geometric characteristics of nanoscale structures in thin films.

**Figure 3:**
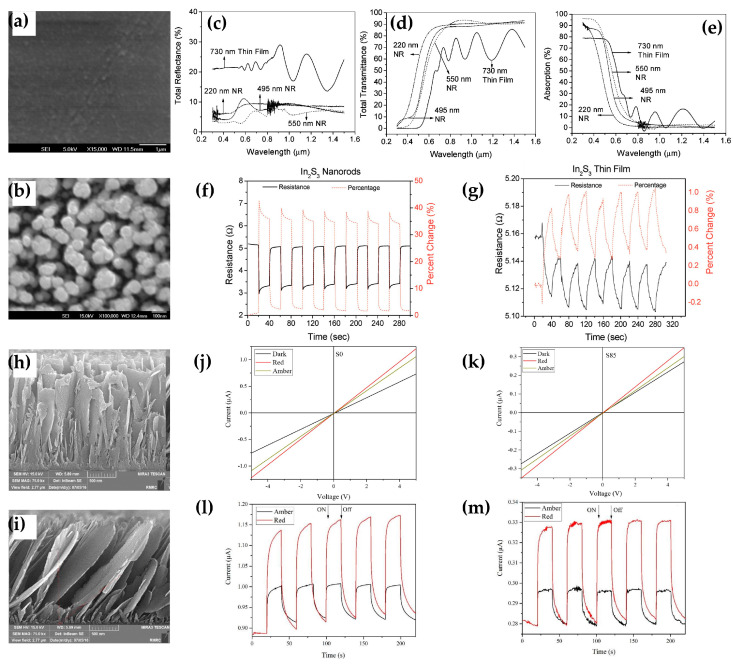
SEM images of In_2_S_3_ grown on a glass substrate (**a**) at vertical incidence and (**b**) at an angle of 85°. Measurement of (**c**) reflectance, (**d**) transmittance, and (**e**) absorbance as a function of the length of NRs. Resistance and resistance percentage change over time for (**f**) NRs and (**g**) TF samples (adapted from [88] with permission from ACS Publications). Cross-sectional SEM images of samples deposited (**h**) at vertical incidence and (**i**) at an angle of 85°. I–V curve of the device deposited (**j**) at vertical incidence and (**k**) at an angle of 85°. Photodetection characteristics of the device deposited (**l**) at vertical incidence and (**m**) at an angle of 85° (adapted from [90] with permission from Elsevier B.V.).

Cansizoglu et al. implemented an array of NR-In_2_S_3_ with excellent light absorption characteristics using thermal evaporation-based OAD technique [88]. The devices were fabricated at a consistent deposition rate of 2 nm·s^−1^ and a rotation speed of 1 rpm at 85°. Figure 3a,b depicts the SEM images of In_2_S_3_ deposited on a glass substrate with a TF structure and an NR structure using the OAD method. The analysis results show that the maximum porosity is 31.1% on the NR substrate. As previously mentioned, this porosity leads to high levels of light absorption, which is an important parameter affecting the performance of photosensitive photodetectors [89]. Subsequently, optical measurements, including reflectance, transmittance, and absorbance, were performed for each device. Figure 3c, Figure 3d, and Figure 3e illustrate reflectance, transmittance, and absorbance spectra, respectively. While the TF device exhibited approximately 20% reflectance, the NR device showed a length-dependent decrease, with the 550 nm NR device demonstrating less than 3% reflectance. Also, at a wavelength of 400 nm, the transmittance in the 550 nm NR device decreased to less than 3%. Furthermore, the transmittance of the TF device was 79%, while the NR device with a length of 550 nm showed an absorption rate of 96% at wavelengths below 500 nm. This can result in excellent photoconductive and photovoltaic properties of In_2_S_3_ NRs deposited via OAD technique. Finally, for photoconductivity testing of NRs and TF—In_2_S_3_ devices, samples were prepared on ITO/glass substrates. Ag films were deposited through sputtering, and the resistance of the samples was measured through the resistor between the Ag layer and the ITO contact layer. As depicted in Figure 3f, the NR device exhibited a maximum 40% reduction in resistance upon the incidence of 465 nm light, thus stabilizing rapidly. In contrast, the TF sample exhibited less than 1% variation, showing a drifting characteristic over time. These results highlight the stability and superior photoconductive response of the NR sample compared to the unstable characteristics of the TF sample (Figure 3g). In this study, although the performance of the device as a photodetector cannot be evaluated, the preliminary photoconductivity results demonstrate the potential excellence of NR-In_2_S_3_ samples fabricated using OAD technique. This performance, based on enhanced light absorption, suggests that it could be an attractive approach for future applications as a photodetector.

As another example, SnS was deposited using thermal evaporation-based OAD technique [90]. SnS was deposited on glass substrates at speeds of 10 Å·s^−1^, both at 0° and 85°. The optical band gap of the SnS sample in this study was estimated using the Tauc plot to be 1.48 eV at 0° and 1.6 eV at 85°. To study the device performance according to the deposition angle, Au metal interdigital electrodes (IDEs) were deposited onto the SnS to prepare the photodetector. Figure 3h,i show the SEM images of devices deposited at 0° and 85°, revealing increased porosity on the surface of the NCs produced through OAD at an 85°. Subsequently, the I-V characteristics of the photodetector were measured under dark conditions and illumination with red light (650 nm, 4.8 mW·cm^−2^) and amber light (595 nm, 1 mW·cm^−2^) under ambient conditions (Figure 3j,k). Both devices exhibited excellent linearity and symmetry in photoresponse due to the ohmic contact formed between the Au electrode and SnS. A greater response was observed in the red light compared to amber due to the difference in light intensity. Furthermore, to examine the photodetection characteristics of the device, a bias voltage of 5 V was applied under dark, red, and amber illumination, and the photo-pulse response was compared through a 20 s on/off switching cycle (Figure 3l,m). Under illumination at both wavelengths, obvious photocurrent was observed, with relatively high photocurrent observed under red light. Consequently, excellent reproducibility and stable characteristics were observed in the NCs-SnS device. To characterize the performance of these photodetectors, traditionally used parameters such as responsivity, detectivity, rise time, and decay time were investigated and recorded in Table 1. NCs-SnS devices exhibited decreased responsivity and detectivity due to their thinner thickness. This can be attributed to the increased resistance caused by the heightened crystal boundaries due to the previously mentioned increased porosity. Crystal boundaries not only act as energy barriers for charge carriers but also significantly increase the probability of electron scattering. However, in terms of photodetection speed, the NCs-SnS device showed significantly shorter response times compared to devices deposited with vertical incidence. This is indicative of the higher sensitivity to illumination for the NCs-SnS device, likely due to the increased trapping of light on the porous surface, enhancing interaction with light. This thermal evaporation facilitates the formation of highly porous nanostructures with excellent light absorption. Nevertheless, many studies report basic photoconductivity but omit key photodetector metrics such as detectivity or EQE, limiting comparability across materials.

Among the studies reviewed in this section, Cansizoglu et al. demonstrated a marked enhancement in light absorption by employing In_2_S_3_ nanorod arrays fabricated via thermal evaporation-based OAD, achieving up to ~96% absorbance at 500 nm [88]. This underscores the efficacy of OAD in tailoring optical responses through nanostructural engineering. However, their evaluation focused primarily on optical and photoconductive behavior, without providing key photodetector performance metrics such as EQE or detectivity, limiting quantitative benchmarking. In contrast, Shahidi et al. implemented a similar OAD approach using SnS films and presented more comprehensive device-level characterization, including responsivity and detectivity [90]. Their devices exhibited shorter response times, yet suffered from lower responsivity and elevated resistance, likely attributed to increased grain boundaries induced by high porosity. Collectively, these studies illustrate a fundamental trade-off inherent in thermal evaporation-based OAD photodetectors: while nanostructuring enhances optical absorption and temporal response, it may simultaneously degrade electrical transport properties due to morphological inhomogeneity. Future work should thus aim to optimize this balance, with attention to process scalability and uniformity across large-area substrates.

### 3.4. OAD Photodetectors Deposited by Sputtering

Sputtering, a deposition technique widely employed in actual industrial settings, offers precise control over stoichiometry during the growth process of films utilizing OAD technique. It boasts advantages such as shape control and the generation of uniform layers. Additionally, it excels in producing highly crystalline films with excellent adhesion at low temperatures for a variety of materials [91,92,93].

**Figure 4:**
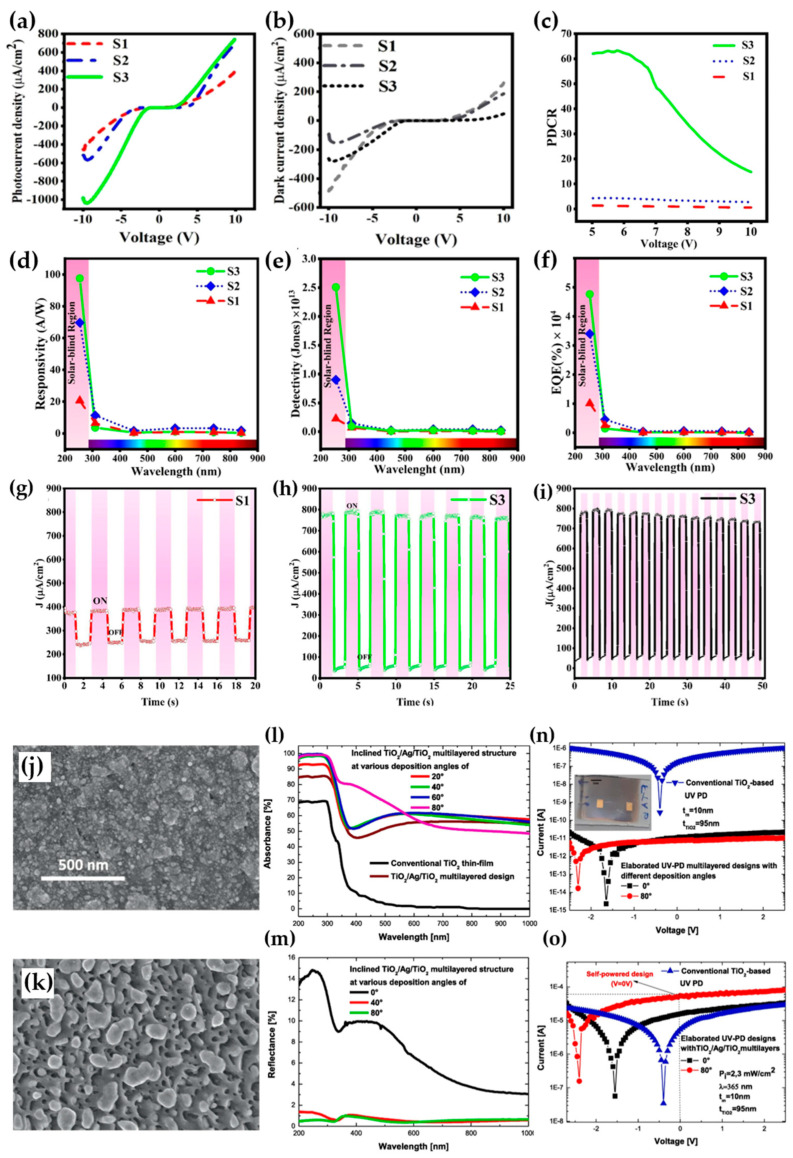
(**a**) Comparison of photocurrent density and (**b**) dark current density in β-Ga_2_O_3_-based PD devices. (**c**) *PDCR* characteristics of the device as a function of voltage. Wavelength-dependent (**d**) responsivity, (**e**) detectivity, and (**f**) EQE comparisons of the device. Photocurrent response characteristics for (**g**) S1 and (**h**) S3 samples (10 V, 254 nm), and (**i**) short-term repeatability measurement of the S3 device (adapted from [94] with permission from ACS Publications). Surface SEM images of devices deposited (**j**) at vertical incidence and (**k**) at an angle of 80°. (**l**) Absorbance and (**m**) reflectance spectrum data of devices fabricated with angles ranging from 0° to 80°. I–V characteristics of devices measured under (**n**) dark and (**o**) UV illumination (adapted from [95] with permission from Elsevier B.V.).

In August 2023, Yaghoubizadeh et al. reported on the impact of controlling the morphological structure through deposition utilizing RF sputtering-based OAD technology, at deposition angles of 0° (S1), 45° (S2), and 65° (S3), on the optical properties of β-Ga_2_O_3_ NRs grown on *p*-Si substrates [94]. The cleaned *p*-Si substrate experienced thermal growth of SiO_2_ through annealing in ambient air at 925 °C. Subsequently, OAD technology was employed to deposit β-Ga_2_O_3_, followed by sputtering of Au to prepare the metal–semiconductor–metal photodetector (MSM-PD) device. Figure 4a and Figure 4b depict the J-V characteristics measured under 254 nm (4.21 mW·cm^−2^) illumination and in the dark, respectively. For samples deposited at higher deposition angles, higher photocurrent density is observed, attributed to light trapping by inclined NRs and carrier trapping at the surface due to oxygen absorption under illumination. The reduction in the density of crystal defects and oxygen vacancies may account for the decrease in current under dark conditions. The decrease in dark current indicates lower leakage current in the samples. Furthermore, the sensitivity of the device was extracted within a bias range of 5~10 V, and in the case of the S3 sample, a remarkably high sensitivity value of up to 63.26 was observed at voltages below 5.7 V (Figure 4c; although the figure is expressed in *PDCR*, the *PDCR* formula in the cited paper is the same as the commonly used sensitivity formula, Equation (1)). To quantitatively evaluate the performance of the photodetector, responsivity, detectivity, and EQE were measured at various wavelengths (Figure 4d–f). Consequently, samples deposited at the highest deposition angle using OAD technique exhibited outstanding capabilities with values of 95.28 A·W^−1^, 2.51 × 10^13^ Jones, and 4.8 × 10^4^%, respectively. Finally, the stability and photoresponse time of the devices fabricated through photo switching were evaluated under 254 nm (7.89 × 10^6^ W·cm^−2^) illumination after applying a 10 V bias. The results revealed a significant improvement in rise and decay times for the S3 sample (0.088 s, 0.044 s) compared to the S1 sample (0.125 s, 0.128 s) (Figure 4g,h). Furthermore, as can be observed in Figure 4i, the S3 sample exhibited excellent performance even in short-term repeatability measurements.

Next, Ferhati et al. proposed a cost-effective TiO_2_/Ag/TiO_2_ nanostructure-based photodetector using DC sputtering-based OAD technique [95]. The devices were fabricated at incident angles of 0°, 20°, 40°, 60°, and 80°, systematically investigating the impact of OAD technique on the optical properties. Figure 4j,k show the SEM image of devices fabricated at incident angles of 0° and 80°. Devices produced through the conventional vertical incidence approach exhibit a high density of films, whereas those fabricated at an 80° angle using OAD display enhance porosity. To assess the influence of this porosity and the insertion of an Ag metal layer on the optical behavior of the device, absorbance and reflectance were examined (Figure 4l,m). The improved absorbance due to the optical trapping effect of the inserted Ag metal layer is evident compared to the traditional TiO_2_ film.

Particularly, the sample deposited at an 80° angle using OAD demonstrates nearly perfect absorbance, exceeding 98% for UV light, thanks to the inclined NCs serving as effective light traps. The significantly reduced reflectance in the spectrum of Figure 4m suggests enhanced absorbance. This combination of OAD and the Ag layer exhibits outstanding potential for UV photodetector applications, based on improved absorbance and reduced surface reflectance. Building upon these results, traditional TiO_2_, vertically deposited TiO_2_ MSM, and TiO_2_ MSM photodetectors deposited at an 80° were fabricated to investigate the photoelectronic performance of the devices under dark and UV illumination (365 nm, 2.3 mW·cm^−2^). Figure 4n,o present the photoelectronic I-V curves of the two devices. The MSM device exhibits extremely low dark current, below 10 pA, attributed to the potential barrier role of the inserted Ag metal layer hindering carrier transmission in the photodetector channel. Furthermore, the negative shift in operating voltage in the MSM structure confirms the excellent self-powering capability of the UV photodetector.

### 3.5. OAD Photodetectors Deposited by Pulsed Laser Deposition

For OAD photodetectors using PLD, one advantage is that the elemental composition of complex or multicomponent target materials can be accurately retained in the deposited film. This enables precise stoichiometric control, which is essential for fabricating high-performance oxide-based devices. Moreover, the deposition characteristics of PLD, characterized by defect-free growth, offer high crystallinity and excellent quality [96]. This is particularly suitable for UV light detection. However, the PLD-based OAD method is inherently limited by its point-source nature, restricting deposition uniformity over wafer-scale areas. This imposes barriers for integration into practical large-scale optoelectronic arrays or CMOS back-end processes.

**Figure 5:**
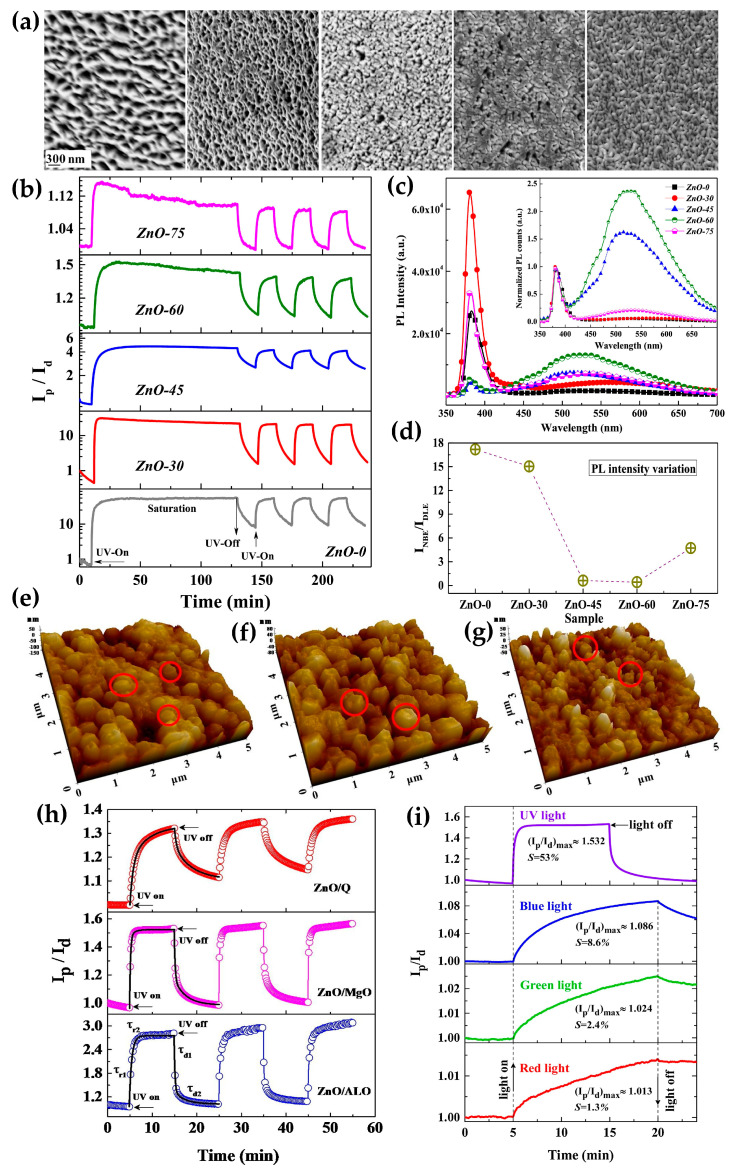
(**a**) Surface SEM images of ZnO samples deposited at various angles (from left to right: 0°, 30°, 45°, 60°, 75°). (**b**) Time-dependent *PDCR* measurements of device under 365 nm illumination. (**c**) PL spectra of ZnO samples grown at different angles, and (**d**) ratio of peak intensities for near-band emission and deep-level emission (adapted from [97] with permission from Elsevier B.V.). AFM images of ZnO nanostructures grown on (**e**) Al_2_O_3_, (**f**) MgO, and (**g**) quartz substrates. (**h**) Photodetection characteristic curves of ZnO samples grown on different substrates. (**i**) *PDCR* characteristics measured under various illuminations for ZnO grown on MgO substrate (adapted from [98] with permission from Elsevier B.V.).

Taking advantage of these characteristics, Soni et al. introduced a catalyst-free self-seeded ZnO photodetector using PLD [97]. ZnO was deposited on a quartz substrate using OAD technique at seeding angles of 0°, 30°, 45°, 60°, and 75°, and the growth using PLD eliminates the possibility of undesirable doping and impurities. Figure 5a presents SEM images corresponding to each growth angle. At a seeding angle of 0°, a clear nanowall (NWL) structure is observed, and as the deposition angle increases, the surface morphology undergoes significant changes. Analysis of porous regions revealed a decrease from a maximum of 31.42% at 0° to a minimum of 9.81% at 75°. To compare the photocurrent characteristics based on these differences in porous areas, photocurrent cycle measurements were conducted under 365 nm illumination for different growth angles (Figure 5b). As a result, at a bias voltage of 1.5 V, the sensitivity of the device increases as the deposition angle decreases, showing a maximum value of 57.67 A·A^−1^ at 0° and a minimum value of 1.16 A·A^−1^ at 75°. Figure 5c shows the photoluminescence (PL) spectra of all ZnO nanostructures, each composed of two distinct emission peaks. In the PL graph, the observed free exciton emission leads to high UV emission intensity, significantly surpassing the visible range luminescence of ZnO-0°, ZnO-30°, and ZnO-75° devices. Furthermore, these devices exhibit faint and broad peaks in the visible light range. In contrast, ZnO-45° and ZnO-60° display intense peaks in the green–red wavelength range, known as Deep Level Emission (DLE), arising from electron transitions related to defects in the grown nanostructures. DLE is typically generated due to various types of defects, primarily oxygen vacancies and impurities like zinc lattice. During PLD, controlling the seeding angle allows for the modulation of defect levels. Near Band Emission (NBE) predominates for samples grown at lower seeding angles (0° and 30°). Lower intensity in visible light emission is desirable in many applications. Therefore, the ratio of the intensity of visible light emission to UV emission (I_NBE_/I_DLE_) is considered an important criterion for indirectly evaluating the crystallinity of ZnO. Figure 5d displays the calculated ratio, confirming the high crystallinity of PLD-grown ZnO nanostructures. Unlike Si-based UV photodetectors, the low sensitivity to visible light in the sample is appealing as it eliminates the need for visible light filtering. This is particularly noteworthy due to the Si-based UV photodetectors requiring visible light filtering due to their strong visible light sensitivity.

Furthermore, Soni et al. utilized PLD-based OAD technique to deposit ZnO at an 85° on Al_2_O_3_, MgO, and quartz substrates, investigating the impact of substrates on the growth [98]. Figure 5e–g display AFM images of ZnO nanostructures grown on each substrate. The surface roughness for Al_2_O_3_, MgO, and quartz substrates is 38.2 nm, 35.4 nm, and 16.8 nm, respectively, providing an effective surface area to enhance photoresponse. Au electrodes were deposited to investigate the photodetection characteristics of the ZnO devices produced using OAD. Figure 5h illustrates the *PDCR* as a function of time characteristics of the fabricated photodetector under 254 nm illumination. The *PDCR* characteristics of the device measured at a bias voltage of 0.1 V demonstrate that devices fabricated on all substrates exhibit significant photocurrent responses. Additionally, to evaluate the device usefulness as a UV detector, the photocurrent of the ZnO/MgO device was measured under red, green, and blue visible light and compared with the UV measurements (Figure 5i). As a result, the device showed sensitivity of less than 10% under various visible light illuminations, while exhibiting a high sensitivity of approximately 53% and excellent rise and decay curves under UV illumination. This suggests the potential of ZnO devices fabricated on all three different substrates to be used as efficient UV photodetectors.

Several studies reviewed in this study have consistently demonstrated the performance benefits of OAD-based nanostructures, including enhanced responsivity and light absorption. These improvements arise from stronger light–matter interaction enabled by the porous and anisotropic morphology formed during oblique deposition. The performance indicators of the photodetectors investigated so far are summarized and displayed in the following Table 2.

## 4. Extended Applications of OAD-Engineered Nanostructures

In the previous section, we discussed OAD photodetectors, which utilize OAD technique to enhance the light absorption and response of photodetectors. In this section, we further expand on the concept of OAD-engineered nanostructures by exploring a wide range of applications (such as anti-reflective (AR) coatings, solar cells, gas sensors, and water splitting), beyond the scope of photodetectors. These applications benefit from tunable optical properties of the nanostructures, modulated through morphological modifications, which can be improve light absorption and overall device performance. Additionally, we investigate how the nanostructures generated by OAD can interact with the photoresponse of other materials to induce functionalized properties, even in cases where these nanostructures do not inherently exhibit useful optical properties.

In AR coatings and solar cells, OAD is employed to either reduce light reflectance, thereby enhancing the amount of light absorbed by the device, or to improve the photoresponse by optimizing light–matter interactions through the formation of porous nanostructures [21,27,99,100,101,102,103,104,105,106,107,108,109,110,111,112,113]. On the other hand, for applications such as gas sensors and water splitting, modifying the film morphology increases the number of active sites where photogenerated carriers can react [114,115,116,117,118,119,120,121,122,123,124,125]. This structural adjustment enhances the surface area and reactivity, facilitating more efficient interactions with gases or ions, which is crucial for improving the performance of these devices.

Recent studies have shown that OAD materials can be effectively utilized in memory and synaptic devices, highlighting their multifunctional potential beyond photodetection. These application devices are garnering significant attention due to the importance in processing large volumes of data in modern society. Nanostructures formed by OAD can induce defect sites, facilitating the generation of active ion recombination and charge trapping effects that are integral to the operating mechanisms of memory and synaptic devices. At the end of this section, we discuss future directions in OAD-based architecture and nanostructure engineering. In particular, we explore the emerging field of photonic memory and synaptic devices, which show great promise as future applications of OAD in photonics [126,127]. Various application cases of OAD technology are introduced in Table 3.

### 4.1. OAD-Engineered Nanostructures for Solar Cell Applications with AR Coating

Solar cells are devices that convert sunlight into electrical signals through the photoelectric effect. To effectively generate electricity from light, several components are essential, including a transparent electrode, an electron transport layer (ETL), a hole transport layer (HTL), AR coatings, and encapsulation, as well as photoresponsive materials that can efficiently generate and transfer photogenerated electron–hole pairs. Among them, AR coatings play a crucial role by enhancing the light absorption efficiency of solar cells, reducing light reflection, and allowing a broader spectrum of wavelengths to penetrate the device [99,101].

**Figure 6:**
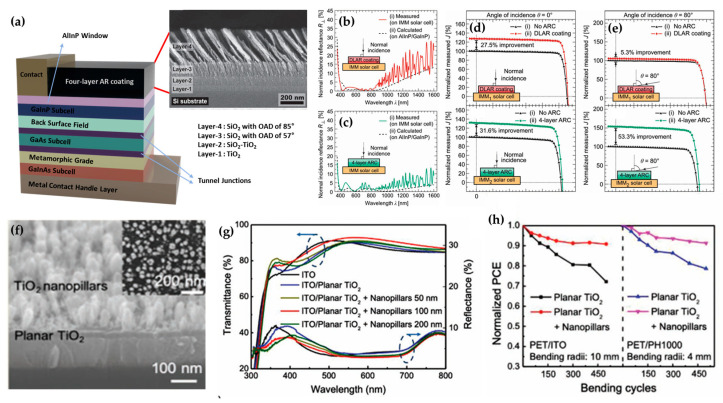
(**a**) Structure of IMM triple-junction solar cell device and cross-sectional SEM image of four-layer AR coating. Measured and calculated normalized incidence reflectance of (**b**) DLAR coating and (**c**) four-layer AR coating. Normalized short-circuit current density in case of (**d**) normal incidence and (**e**) oblique incidence (adapted from [110] with permission from Wiley). (**f**) Cross-sectional and top-view images of planar TiO_2_ and TiO_2_ NPs. (**g**) Transmittance spectra of planar TiO_2_ and TiO_2_ NPs films on ITO. (**h**) Normalized PCE of PSC with and without TiO_2_ NPs depending on bending cycles (adapted from [112] with permission from Wiley).

In 2013, Yan et al. presented an inverted metamorphic (IMM) triple-junction solar cell device using a four-layer AR coating [110]. By employing a genetic algorithm (GA), they optimized the AR coating with nanoporous thin films, comparing it to a double-layer AR (DLAR) coating. Figure 6a shows the structure of the solar cell and a cross-sectional SEM image of the four-layer AR coating. The DLAR coating consisted of TiO_2_ and SiO_2_ layers, whereas the four-layer AR coating featured two SiO_2_ layers, with deposition angles of 57° and 85°, respectively. In particular, the two SiO_2_ OAD layers, with refractive indices of 1.32 and 1.11, showed that the layers deposited at larger angles exhibited lower refractive indices. In Figure 6b,c, the four-layer AR coating demonstrated a measured wavelength-averaged reflectance of 3.9%, which is lower than the 7.9% reflectance of the DLAR coating. These results suggest that the nanostructures generated by OAD enhanced broadband AR effects, leading to significant improvements in short-circuit current density in the IMM triple-junction solar cell device with the four-layer AR coating, as shown in Figure 6d,e. This underscores the importance of depositing two OAD layers at different angles to enhance the AR performance of solar cells.

To improve the performance of solar cells, the strategy of alternating materials with different refractive indices to form a complementary multilayer AR coating is essential for minimizing reflection across a broad spectral range [128,129]. Particularly, OAD is an attractive advantageous approach, as it enables the adjustment of the refractive index of materials, offering flexibility in designing tailored AR coatings. Furthermore, it is a low temperature process that can minimize the thermal impact on solar cells, making it highly appropriate for flexible solar cell applications.

For example, mesoporous TiO_2_ has traditionally been considered the most effective ETL in flexible perovskite solar cells (PSCs). However, its application faced certain limitations due to the poor thermal stability of flexible substrates, such as polymers, which require the mesoporous TiO_2_ to be fabricated at high temperatures (around 500 °C). In 2021, Wu et al. introduced a new approach using planar TiO_2_/TiO_2_ nanopillar (NP) bilayers as the ETL [112]. As shown in Figure 6f, the TiO_2_ NPs were formed through an oxygen-assisted deposition (OAD) technique on a planar TiO_2_ layer, which was conventionally deposited by e-beam evaporation. The resulting TiO_2_ NPs improved the AR performance, exhibiting the highest transmittance and the lowest reflectance at a nanopillar thickness of 100 nm (Figure 6g). The average power conversion efficiencies (PCEs) of 18 devices with the structure were 16.33 ± 0.48% with TiO_2_ nanopillars, compared to 14.72 ± 0.42% without them. Notably, the TiO_2_ NPs demonstrated excellent stability, maintaining a stable PCE after 500 bending cycles, in contrast to the PSC device without the TiO_2_ NPs (Figure 6h). The flexible PSCs were successfully fabricated with TiO_2_ NPs, which exhibited both AR properties and the advantage of low-temperature OAD fabrication. However, there are still areas where further improvements need to be made for these applications. Although AR coatings fabricated by OAD significantly improve light trapping, their integration into commercial solar modules necessitates large-area compatibility and mechanical robustness. Current OAD-based AR structures are often tested on small samples, and their adhesion and optical stability under long-term outdoor exposure remain underexplored.

### 4.2. OAD-Engineered Nanostructures for Memory/Synaptic Devices

**Figure 7:**
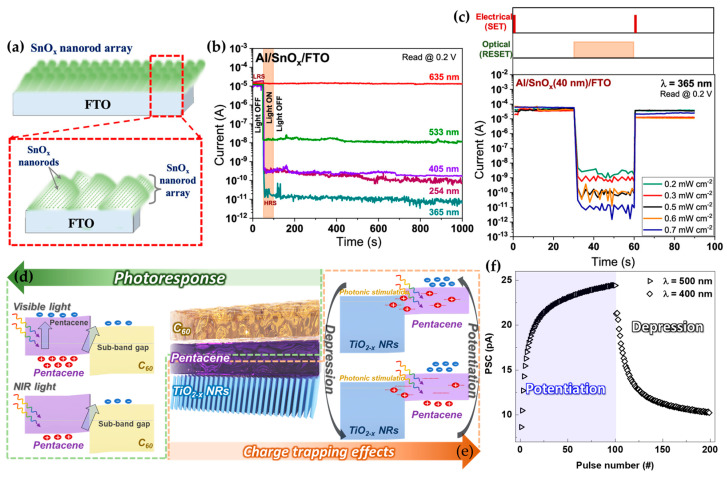
(**a**) Cross-sectional illustration of SnO_x_ NRs array on FTO. (**b**) Resistive switching characteristics depending on light with different wavelengths. (**c**) Resistance switching characteristics stimulated by 365 nm UV light at different power densities (adapted from [126] with permission from ACS Publications). (**d**) Photoresponse mechanism of synaptic device. (**e**) Potentiation and depression from charge trapping effects. (**f**) Potentiation–depression characteristic by light wavelength modulation from 500 nm to 400 nm (adapted from [127] with permission from Wiley).

In 2023, S. P. et al. proposed an optical memristor device using SnO_x_ NRs fabricated through an OAD process at an 80° tilt [123]. The OAD method results in nanorods with a high concentration of oxygen vacancies due to the slow deposition rate and low energy during growth, which prevents the formation of the SnO_2_ phase. As a result, the SnO_x_ NRs have an increased percentage of oxygen vacancies. These vacancies are critical for the device’s memristor behavior. The memristor structure features SnO_x_ as a wide-band gap layer sandwiched between an FTO substrate and an Al top electrode (Figure 7a). When a positive voltage (SET) is applied, Sn-O bonds form, creating conductive filaments that switch the device from a high-resistance state (HRS) to a low-resistance state (LRS). Conversely, a negative voltage (RESET) breaks these bonds, returning the device to HRS. Notably, the device also achieves RESET behavior under UV or visible light illumination, eliminating the need for a negative voltage (Figure 7b). This optical RESET mechanism exploits the photoexcitation of oxygen ions within SnO_x_ NRs, which recombine with oxygen vacancies, thereby destroying conductive filaments and achieving higher HRS levels. The I_LRS_/I_HRS_ ratio under light (~10^6^ at 365 nm) significantly outperforms the ratio achieved through electrical RESET (~10^2^), demonstrating superior performance. Additionally, Figure 7c shows that multiple states can be distinguished depending on light incidence, with the current decrease becoming more significant as the intensity increases. The optical method also offers additional advantages, such as lower operating voltages and the ability to retain the HRS even after the light is removed. Furthermore, the intensity of the light can modulate the resistance, enabling multiple distinct states. The study highlights the impact of OAD in achieving high oxygen vacancy concentrations and demonstrates the importance of these vacancies in optimizing memristor performance through enhanced resistive switching and higher I_LRS_/I_HRS_ ratios.

In 2024, Jeon et al. demonstrated a photosynaptic device utilizing OAD [124]. The ternary heterostructure, composed of functionalized TiO_2−x_ NRs/pentacene and pentacene/C_60_ junctions, exhibits charge trapping effects and broadband photoresponses spanning from visible to near-infrared (NIR) light, respectively. Specifically, pentacene, with a band gap of 1.78 eV, shows a photoresponse to light in the 400 nm to 700 nm range, while the sub-band gap of 1.2 eV extends the photoresponse to up to 800 nm (Figure 7d). Based on these photoresponses, the internal and interface charge trapping effects for the synaptic characteristics are implemented by pentacene and TiO_2−x_ NRs. As shown in Figure 7e, the trap sites within pentacene exhibit synaptic characteristics when stimulated by shorter wavelengths (500 nm), whereas the TiO_2−x_ NRs/pentacene junction induces excitatory postsynaptic current (EPSC) behaviors under light stimulation from 500 nm to 800 nm. To further investigate the charge trapping mechanism, they conducted contact angle measurements, which revealed a surface energy of 26.75 mN·m^−1^ for TiO_2−x_ thin films without OAD, and a significantly higher value of 39.91 mN·m^−1^ for TiO_2−x_ NRs. This increase in hydrophilicity of TiO_2−x_ NR surfaces provide more trap sites when forming a heterojunction with pentacene. As a result, the potentiation–depression curve, as shown in Figure 7f, was observed for the ternary heterostructure. Notably, potentiation induced by 500 nm photonic stimulation transitions to depression upon 400 nm photonic stimulation with higher energy, leading to a progressive detrapping process. Furthermore, they fabricated a device with the same structure on a flexible paper substrate. As a result, they demonstrated excellent synaptic plasticity control even after 1000 bending tests. These results demonstrate the potential of OAD technique for flexible substrate applications. This study underscores how the interaction between photogenerated carriers and nanostructures formed via OAD underpins the photosynaptic characteristics of the device through the trapping and detrapping processes. As such, recent research on OAD-based optoelectronic devices has focused on implementing memory and synaptic devices through defects intentionally created by nanostructures.

In 2025, Lee et al. introduced a synaptic transistor based on organic semiconductors fabricated via OAD [130]. OAD technique applied to the deposition of organic semiconductor DNTTs induces photoinduced traps in the film by implementing nanostructures. Compared to conventional planar structures, the unique particle arrangement and interface defect density are increased. These characteristics are advantageous for optical synaptic transistors whose conductance is controlled by light stimulation. A notable feature is the possibility of simultaneous multi-element stimulation. In order to expand from a single device to a circuit, multiple operations are essential. This study demonstrates the possibility of scaling by demonstrating the simultaneous activation of multiple OADs.

Generally, defect states present within semiconductors or at their interfaces are considered factors that can lead to performance degradation and reduced lifespan of optoelectronic devices [131,132]. To address these issues, efforts have been made to form defect-free films with single-crystal structures or to improve interfaces through appropriate treatments. However, the OAD-based electronic devices introduced in this review achieve their functionality by intentionally creating nanostructures with abundant defects. These defects enhance optical properties, enable charge trapping, or facilitate filament formation, thereby increasing the number of reactive sites induced by morphological changes.

Light, with advantages such as fast response speed, non-contact input, and the photoelectric effect, has been one of the most actively researched new inputs for enhancing the multifunctionality of electronic devices [133,134]. Beyond the two-terminal structures such as photodetectors, memory, and synaptic devices discussed earlier, research is continuously expanding into more diverse application fields. From this perspective, we believe that the improvement in optical properties based on OAD technology is a simple yet efficient method that has potential applications in the development of advanced optical-based electronic devices such as floating gate memory and optoelectronic logic circuits. Beyond photodetection, OAD-engineered nanostructures have shown potential in emerging device concepts, including neuromorphic systems and synaptic electronics due to their geometry-dependent optical and electrical behaviors.

## 5. Conclusions

This review has provided a comprehensive exploration of photodetectors utilizing OAD technique. The capability of OAD to engineer unique nanostructures, such as porous columns and nanorods, allows for the precise manipulation of optical properties, including reflectance, absorption, and transmission. These advantages have established OAD as a key method in the development of high-performance photodetectors and light-sensitive devices. OAD technology continues to advance, overcoming challenges and creating new opportunities in nanostructure design. Recent progress in controlling porosity, morphology, and scalability has expanded its applications in diverse fields, including optoelectronics, energy harvesting, and biosensing. Moreover, with emerging technologies like photonic memory and artificial synapses gaining momentum, OAD-engineered structures are well positioned to contribute significantly to their development. In particular, this review presents how OAD-induced nanostructures can be controlled through deposition conditions to modulate optical properties such as light absorption, reflectance, and responsivity. Furthermore, by classifying photodetectors based on four representative PVD techniques, we provide insight into the correlation between the deposition method, nanostructure morphology, and device performance. Finally, we discuss future research directions, including multifunctional applications of OAD in neuromorphic and wearable optoelectronics, highlighting both its current strengths and remaining challenges.

Despite its promise, several challenges must be addressed for OAD to achieve broader practical application. These include difficulties in achieving uniform nanostructures over large areas due to angle-dependent deposition, limited compatibility with standard CMOS fabrication processes, and constraints in applicable materials. Future research directions may involve combining OAD with complementary techniques for hybrid nanostructure fabrication, as well as extending OAD photodetector to neuromorphic or optical synaptic devices. Additionally, the use of OAD in flexible and wearable optoelectronics holds potential for next-generation applications.

## Figures and Tables

**Table 1:** Photodetection parameters of the SnS device.

Wavelength	Deposited Angle	R (AW^−1^)	$D^{*}$ (Jones)	τ_r_ (s)	τ_d_ (s)
595 nm (Amber)	0°	2.46 × 10^−3^	8.38 × 10^6^	2.15	12.21
	85°	0.46 × 10^−3^	4.99 × 10^6^	0.69	8.48
650 nm (Red)	0°	1.45 × 10^−3^	2.58 × 10^6^	3.59	11.18
	85°	0.29 × 10^−3^	1.78 × 10^6^	1.49	8.77

**Table 2:** Comprehensive analysis of OAD photodetectors.

Material	Structure	Deposition Angle (°)	Porosity (%)	Deposition Technique	Detecting Wavelength (nm)	R (A·W^−1^)	$D^{*}$ (Jones)	τr (s)	τr (s)	Year	Ref
TiO_2_	Nanowire	85	-	E-beam evaporation	300~900	-	-	-	-	2022	[83]
SnO_2_	Nanowire	85	-	E-beam evaporation	370	0.36 × 10^−3^	3.02 × 10^9^	0.72	1.78	2019	[84]
β-Ga_2_O_3_	Nanorod	65	-	Sputtering	254	95.28	2.51 × 10^13^	0.09	0.04	2023	[88]
TiO_2_	Nano sculpture	80	-	Sputtering	365	205 × 10^−3^	5.3 × 10^13^	-	-	2020	[90]
ZnO	Nano structure	0~85	~31.42	PLD	365	-	-	-	-	2024	[94]
ZnO	Nano structure	85	~26.32	PLD	254	-	-	9	7	2020	[95]
In_2_S_3_	Nanorod	85	31.1	Thermal evaporation	465	-	-	-	-	2010	[97]
SnS	Nanoplate	85	-	Thermal evaporation	595, 650	0.46 × 10^−3^, 0.29 × 10^−3^	4.99 × 10^6^, 1.78 × 10^6^	0.69, 1.49	8.48, 8.77	2020	[98]

**Table 3:** Various applications of OAD-engineered nanostructures.

Application	Material	Deposition Technique	Deposition Angle (°)	Year	Ref.
AR coating	ITO	E-beam evaporation	85	2021	[21]
	SiO_2_	E-beam evaporation	85	2024	[99]
	MgF_2_	E-beam evaporation	0–86	2024	[100]
	MgF_2_	E-beam evaporation	-	2015	[101]
	TiO_2_	E-beam evaporation	86	2021	[102]
	HfO_2_	E-beam evaporation	88	2017	[103]
	SiO_2_	E-beam evaporation	30–80	2021	[104]
	SiO_2_	Sputtering	67	2021	[105]
	Ag	Sputtering	5–60	2016	[106]
Solar cell	CdTe	Sputtering	20–80	2020	[27]
	SnO_x_	Sputtering	80	2023	[107]
	SiO_2_	E-beam evaporation	-	2024	[108]
	Au	E-beam evaporation	80	2014	[109]
	SiO_2_	E-beam evaporation	57, 85	2013	[110]
	Bi	Thermal evaporation	-	2020	[111]
	TiO_2_	E-beam evaporation	85	2021	[112]
	TiO_2_	Sputtering	-	2007	[113]
Gas sensor	In_2_O_3_	Sputtering	85	2023	[114]
	In_2_O_3_	Sputtering	85	2023	[115]
	In_2_O_3_	E-beam evaporation	85	2025	[116]
	WO_3_	Sputtering	85	2013	[117]
Water splitting	ZnO	Pulsed laser deposition	86	2009	[118]
	TiO_2_	E-beam evaporation	86	2009	[119]
	ITO	Sputtering	70	2016	[120]
	Ag	Thermal evaporation	85	2024	[121]
	Co_3_O_4_, NiO	E-beam evaporation	85	2024	[122]
	Ta_3_N_5_	Sputtering	85	2024	[123]
	WO_3_	Sputtering	-	2015	[124]
	TiO_2_	E-beam evaporation	85	2024	[125]
Memory/ synaptic device	SnO_x_	E-beam evaporation	80	2023	[126]
	TiO_2−x_	E-beam evaporation	70	2024	[127]

## Data Availability

Not applicable.

## References

[1] Pan X., Bai L., Wang H., Wu Q., Wang H., Liu S., Xu B., Shi X., Liu H. (2018). Metal–organic-framework-derived carbon nanostructure augmented sonodynamic cancer therapy. Adv. Mater..

[2] Deng Z., Jiang H., Hu Y., Liu Y., Zhang L., Liu H., Li C. (2017). 3D Ordered Macroporous MoS_2_@C Nanostructure for Flexible Li-Ion Batteries. Adv. Mater..

[3] Zhou K., Wang H., Jiu J., Liu J., Yan H., Suganuma K. (2018). Polyaniline films with modified nanostructure for bifunctional flexible multicolor electrochromic and supercapacitor applications. Chem. Eng. J..

[4] Jeon Y., Seo J., Yoo H. (2023). Air-stable ambipolar charge transport behaviors of organic-inorganic hybrid bilayer and application to Au nanoparticle-based floating gate memory. J. Alloys Compd..

[5] Garratt E., Prete P., Lovergine N., Nikoobakht B. (2017). Observation and Impact of a “Surface Skin Effect” on Lateral Growth of Nanocrystals. J. Phys. Chem. C.

[6] Miccoli I., Prete P., Lovergine N. (2015). Mass-transport driven growth dynamics of AlGaAs shells deposited around dense GaAs nanowires by metalorganic vapor phase epitaxy. CrystEngComm.

[7] Lovergine N., Liaci L., Ganière J.D., Leo G., Drigo A., Romanato F., Mancini A.M., Vasanelli L. (1995). Inhomogeneous strain relaxation and defect distribution of ZnTe layers deposited on (100)GaAs by metalorganic vapor phase epitaxy. J. Appl. Phys..

[8] Barranco A., Borras A., Gonzalez-Elipe A.R., Palmero A. (2016). Perspectives on oblique angle deposition of thin films: From fundamentals to devices. Prog. Mater. Sci..

[9] Pyun M.W., Kim E.J., Yoo D.-H., Hahn S.H. (2010). Oblique angle deposition of TiO_2_ thin films prepared by electron-beam evaporation. Appl. Surf. Sci..

[10] Mark A.G., Gibbs J.G., Lee T.-C., Fischer P. (2013). Hybrid nanocolloids with programmed three-dimensional shape and material composition. Nat. Mater..

[11] Lintymer J., Gavoille J., Martin N., Takadoum J. (2003). Glancing angle deposition to modify microstructure and properties of sputter deposited chromium thin films. Surf. Coat. Technol..

[12] Kesapragada S., Victor P., Nalamasu O., Gall D. (2006). Nanospring pressure sensors grown by glancing angle deposition. Nano Lett..

[13] Gao Z., Zhang T., Wang Q., Mayrhofer P.H. (2023). Nanostructured zig-zag γ-Mo_2_N thin films produced by glancing angle deposition for flexible symmetrical solid-state supercapacitors. Mater. Des..

[14] Huang Z., Liu J. (2017). Chiroptically active metallic nanohelices with helical anisotropy. Small.

[15] Chen L., Andrea L., Timalsina Y.P., Wang G.-C., Lu T.-M. (2013). Engineering epitaxial-nanospiral metal films using dynamic oblique angle deposition. Cryst. Growth Des..

[16] Cansizoglu H., Cansizoglu M.F., Watanabe F., Karabacak T. (2014). Enhanced photocurrent and dynamic response in vertically aligned In_2_S_3_/Ag core/shell nanorod array photoconductive devices. ACS Appl. Mater. Interfaces.

[17] Smith W., Zhao Y. (2008). Enhanced photocatalytic activity by aligned WO_3_/TiO_2_ two-layer nanorod arrays. J. Phys. Chem. C.

[18] Kwon H., Sung J.H., Lee Y., Jo M.-H., Kim J.K. (2018). Wavelength-dependent visible light response in vertically aligned nanohelical TiO_2_-based Schottky diodes. Appl. Phys. Lett..

[19] Maudet F., Lacroix B., Santos A.J., Paumier F., Paraillous M., Hurand S., Corvisier A., Marsal C., Giroire B., Dupeyrat C. (2020). Optical and nanostructural insights of oblique angle deposited layers applied for photonic coatings. Appl. Surf. Sci..

[20] Tajik N., Ehsani M., Moghadam R.Z., Dizaji H.R. (2018). Effect of GLAD technique on optical properties of ZnS multilayer antireflection coatings. Mater. Res. Bull..

[21] Chaikeeree T., Mungkung N., Kasayapanand N., Lertvanithphol T., Nakajima H., Horprathum M. (2021). Characterization broadband omnidirectional antireflection ITO nanorod films coating. Opt. Mater..

[22] Kim S.H., Lee S.H., Yu J.S. (2019). Broadband and antireflective characteristics of glancing angle deposited titanium dioxide nanostructures for photovoltaic applications. Thin Solid Films.

[23] Moon H.G., Han S.D., Kang M.-G., Jung W.-S., Kwon B., Kim C., Lee T., Lee S., Baek S.-H., Kim J.-S. (2016). Glancing angle deposited WO_3_ nanostructures for enhanced sensitivity and selectivity to NO_2_ in gas mixture. Sens. Actuators B Chem..

[24] Singh A., Sharma A., Tomar M., Gupta V. (2018). Growth of highly porous ZnO nanostructures for carbon monoxide gas sensing. Surf. Coat. Technol..

[25] Martín M., Salazar P., Álvarez R., Palmero A., López-Santos C., González-Mora J.L., González-Elipe A.R. (2017). Cholesterol biosensing with a polydopamine-modified nanostructured platinum electrode prepared by oblique angle physical vacuum deposition. Sens. Actuators B Chem..

[26] Zhang N., Su X., Free P., Zhou X., Neoh K.G., Teng J., Knoll W. (2013). Plasmonic metal nanostructure array by glancing angle deposition for biosensing application. Sens. Actuators B Chem..

[27] Adhikari D., Junda M.M., Bastola E., Koirala P., Ellingson R.J., Collins R.W., Podraza N.J. (2020). Glancing angle deposited CdTe: Nanostructured films and impact on solar cell performance. Surf. Coat. Technol..

[28] Hu Z., García-Martín J.M., Li Y., Billot L., Sun B., Fresno F., Garcia-Martin A., González M.U., Aigouy L., Chen Z. (2020). TiO_2_ nanocolumn arrays for more efficient and stable perovskite solar cells. ACS Appl. Mater. Interfaces.

[29] Kwon H., Ham J., Kim D.Y., Oh S.J., Lee S., Oh S.H., Schubert E.F., Lim K.G., Lee T.W., Kim S. (2014). Three-Dimensional Nanostructured Indium-Tin-Oxide Electrodes for Enhanced Performance of Bulk Heterojunction Organic Solar Cells. Adv. Energy Mater..

[30] Zhen C., Wu T., Chen R., Wang L., Liu G., Cheng H.-M. (2019). Strategies for modifying TiO_2_ based electron transport layers to boost perovskite solar cells. ACS Sustain. Chem. Eng..

[31] Chen H., Wang P., Wang X., Wang X., Rao L., Qian Y., Yin H., Hou X., Ye H., Zhou G. (2021). 3D InGaN nanowire arrays on oblique pyramid-textured Si (311) for light trapping and solar water splitting enhancement. Nano Energy.

[32] Limwichean S., Kasayapanand N., Ponchio C., Nakajima H., Patthanasettakul V., Eiamchai P., Meng G., Horprathum M. (2021). Morphology-controlled fabrication of nanostructured WO_3_ thin films by magnetron sputtering with glancing angle deposition for enhanced efficiency photo-electrochemical water splitting. Ceram. Int..

[33] Kim J.H., Choi I.Y., Kim J.H., Kim J., Kim Y.K., Kim J.K., Lee J.S. (2021). ZnFe_2_O_4_ Dendrite/SnO_2_ Helix 3D Hetero-Structure Photoanodes for Enhanced Photoelectrochemical Water Splitting: Triple Functions of SnO_2_ Nanohelix. Small.

[34] Xie F., Lu H., Xiu X., Chen D., Han P., Zhang R., Zheng Y. (2011). Low dark current and internal gain mechanism of GaN MSM photodetectors fabricated on bulk GaN substrate. Solid-State Electron..

[35] Ahmed A.A., Devarajan M., Afzal N. (2017). Fabrication and characterization of high performance MSM UV photodetector based on NiO film. Sens. Actuators A Phys..

[36] Oh S., Kim C.-K., Kim J. (2017). High responsivity β-Ga_2_O_3_ metal–semiconductor–metal solar-blind photodetectors with ultraviolet transparent graphene electrodes. ACS Photonics.

[37] Choi W., Park T., Yoo H., Hur J. (2023). Vertical asymmetric metal-semiconductor-metal photodiode based on β-Ga_2_O_3_ thin films fabricated via solution process for arc discharge detection. J. Alloys Compd..

[38] Ishii A., Miyasaka T. (2020). Direct detection of circular polarized light in helical 1D perovskite-based photodiode. Sci. Adv..

[39] Lischke S., Peczek A., Morgan J., Sun K., Steckler D., Yamamoto Y., Korndörfer F., Mai C., Marschmeyer S., Fraschke M. (2021). Ultra-fast germanium photodiode with 3-dB bandwidth of 265 GHz. Nat. Photonics.

[40] Shin J., Kim S., Jang B.C., Yoo H. (2023). Dielectric surface-dependent photogating phenomenon in C8-BTBT leading to broad spectral ultraviolet to near-infrared photoresponse and linearly-weighted synaptic phototransistors. Dyes Pigments.

[41] Kim S., Hong S., Yoo H. (2021). Location-dependent multi-parameter detection behaviors using hetero-interfaced organic anti-ambipolar phototransistors. Sens. Actuators A Phys..

[42] Jiang J., Hu W., Xie D., Yang J., He J., Gao Y., Wan Q. (2019). 2D electric-double-layer phototransistor for photoelectronic and spatiotemporal hybrid neuromorphic integration. Nanoscale.

[43] Pedapudi M.C., Dhar J.C. (2022). Ultrasensitive p-n junction UV-C photodetector based on p-Si/β-Ga_2_O_3_ nanowire arrays. Sens. Actuators A Phys..

[44] Meitei P.N., Singh N.K. (2023). Self-powered photodetector based on a Ag nanoparticle-decorated Gd_2_O_3_ nanorod. ACS Appl. Electron. Mater..

[45] Castillo-Seoane J., Contreras-Bernal L., Obrero-Perez J.M., García-Casas X., Lorenzo-Lázaro F., Aparicio F.J., Lopez-Santos C., Rojas T.C., Anta J.A., Borras A. (2022). Highly Anisotropic Organometal Halide Perovskite Nanowalls Grown by Glancing-Angle Deposition. Adv. Mater..

[46] Jensen M.O., Brett M.J. (2005). Periodically structured glancing angle deposition thin films. IEEE Trans. Nanotechnol..

[47] Wang S., Xia G., He H., Yi K., Shao J., Fan Z. (2007). Structural and optical properties of nanostructured TiO_2_ thin films fabricated by glancing angle deposition. J. Alloys Compd..

[48] Hahn N.T., Ye H., Flaherty D.W., Bard A.J., Mullins C.B. (2010). Reactive ballistic deposition of α-Fe_2_O_3_ thin films for photoelectrochemical water oxidation. ACS Nano.

[49] Flaherty D.W., Dohnálek Z., Dohnálková A., Arey B.W., McCready D.E., Ponnusamy N., Mullins C.B., Kay B.D. (2007). Reactive ballistic deposition of porous TiO_2_ films: Growth and characterization. J. Phys. Chem. C.

[50] He Y., Basnet P., Murph S.E.H., Zhao Y. (2013). Ag nanoparticle embedded TiO_2_ composite nanorod arrays fabricated by oblique angle deposition: Toward plasmonic photocatalysis. ACS Appl. Mater. Interfaces.

[51] Chen C.-Y., Huang J.-H., Song J., Zhou Y., Lin L., Huang P.-C., Zhang Y., Liu C.-P., He J.-H., Wang Z.L. (2011). Anisotropic outputs of a nanogenerator from oblique-aligned ZnO nanowire arrays. ACS Nano.

[52] Kannan V., Inamdar A.I., Pawar S.M., Kim H.-S., Park H.-C., Kim H., Im H., Chae Y.S. (2016). Facile route to NiO nanostructured electrode grown by oblique angle deposition technique for supercapacitors. ACS Appl. Mater. Interfaces.

[53] Han J.H., Kim D., Kim J., Kim G., Fischer P., Jeong H.H. (2023). Plasmonic nanostructure engineering with shadow growth. Adv. Mater..

[54] Wang T., Dong P., Zhu C., Sha P., Gao W., Wu Y., Wu X. (2021). Trace detection of anthrax protective antigens via a competitive method based on surface-enhanced Raman scattering. Sens. Actuators B Chem..

[55] Chu H.O., Song S., Li C., Gibson D. (2017). Surface enhanced Raman scattering substrates made by oblique angle deposition: Methods and applications. Coatings.

[56] Hang Z.Y., Thompson C.V. (2014). Effects of oblique-angle deposition on intrinsic stress evolution during polycrystalline film growth. Acta Mater..

[57] Yadav S., Senapati S., Kumar S., Gahlaut S.K., Singh J.P. (2022). GLAD based advanced nanostructures for diversified biosensing applications: Recent progress. Biosensors.

[58] Poxson D.J., Mont F.W., Schubert M.F., Kim J.K., Schubert E.F. (2008). Quantification of porosity and deposition rate of nanoporous films grown by oblique-angle deposition. Appl. Phys. Lett..

[59] Ye D., Karabacak T., Lim B., Wang G., Lu T. (2004). Growth of uniformly aligned nanorod arrays by oblique angle deposition with two-phasesubstrate rotation. Nanotechnology.

[60] Alvarez R., Garcia-Martin J.M., Lopez-Santos M.C., Rico V., Ferrer F.J., Cotrino J., Gonzalez-Elipe A.R., Palmero A. (2014). On the deposition rates of magnetron sputtered thin films at oblique angles. Plasma Process. Polym..

[61] Chowdhury N., Bedanta S. (2014). Controlling the anisotropy and domain structure with oblique deposition and substrate rotation. AIP Adv..

[62] Xu X., Huang H., Jin L., Wen T., Liao Y., Tang X., Li Y., Zhong Z. (2023). Micromorphology and uniaxial magnetic anisotropy of oblique-sputtered Ni_80_Fe_20_ films on periodically rippled Al_2_O_3_ substrates. Surf. Interfaces.

[63] Xiao X., Dong G., Shao J., He H., Fan Z. (2010). Optical and electrical properties of SnO_2_: Sb thin films deposited by oblique angle deposition. Appl. Surf. Sci..

[64] He Y., Fu J., Zhao Y. (2014). Oblique angle deposition and its applications in plasmonics. Front. Phys..

[65] Starke R., Schober G. (2018). Why history matters: Ab initio rederivation of Fresnel equations confirms microscopic theory of refractive index. Optik.

[66] Hung K.-Y., Liao J.-C. (2008). The application of Fresnel equations and anti-reflection technology to improve inclined exposure interface reflection and develop a key component needed for Blu-ray DVD-micro-mirrors. J. Micromech. Microeng..

[67] Zhou Y., Chan K.K., Lai T., Tang S. (2012). Characterizing refractive index and thickness of biological tissues using combined multiphoton microscopy and optical coherence tomography. Biomed. Opt. Express.

[68] Xiao G.Z., Adnet A., Zhang Z., Sun F.G., Grover C.P. (2005). Monitoring changes in the refractive index of gases by means of a fiber optic Fabry-Perot interferometer sensor. Sens. Actuators A Phys..

[69] Sobahan K., Park Y.J., Kim J.J., Shin Y.S., Kim J.B., Hwangbo C.K. (2010). Nanostructured optical thin films fabricated by oblique angle deposition. Adv. Nat. Sci. Nanosci. Nanotechnol..

[70] Parra-Barranco J., Garcia-Garcia F.J., Rico V., Borras A., Lopez-Santos C., Frutos F., Barranco A., Gonzalez-Elipe A.R. (2015). Anisotropic in-plane conductivity and dichroic gold plasmon resonance in plasma-assisted ITO thin films e-beam-evaporated at oblique angles. ACS Appl. Mater. Interfaces.

[71] Oh M.-K., Shin Y.-S., Lee C.-L., De R., Kang H., Yu N.E., Kim B.H., Kim J.H., Yang J.-K. (2015). Morphological and SERS properties of silver nanorod array films fabricated by oblique thermal evaporation at various substrate temperatures. Nanoscale Res. Lett..

[72] Hurand S., Corvisier A., Lacroix B., Santos A.J., Maudet F., Dupeyrat C., Roja R.G., Morales F.M., Girardeau T., Paumier F. (2022). Anisotropic optical properties of indium tin oxide thin films prepared by ion beam sputtering under oblique angle deposition. Appl. Surf. Sci..

[73] Aiempanakit C., Aiempanakit K. (2022). Structural development and phase transformation behavior of thermally-oxidization Ti by sputtering power and OAD technique. Mater. Chem. Phys..

[74] Qi Z., Tang J., Huang J., Zemlyanov D., Pol V.G., Wang H. (2019). Li_2_MnO_3_ thin films with tilted domain structure as cathode for Li-ion batteries. ACS Appl. Electron. Mater..

[75] Lamichhane S., Sharma S., Tomar M., Chowdhuri A. (2023). Studies on photovoltaic properties of BFO/WO_3_ bilayer thin films for solar energy harvesting applications. Results Opt..

[76] Lee S., Park T., Hur J., Yoo H. (2022). Calcium titanate orthorhombic perovskite-nickel oxide solar-blind UVC photodetectors with unprecedented long-term stability exceeding 500 days and their applications to real-time flame detection. ACS Photonics.

[77] Yang D., Ma D. (2019). Development of organic semiconductor photodetectors: From mechanism to applications. Adv. Opt. Mater..

[78] Zhang Y., Ma Y., Wang Y., Zhang X., Zuo C., Shen L., Ding L. (2021). Lead-free perovskite photodetectors: Progress, challenges, and opportunities. Adv. Mater..

[79] Wu D., Guo J., Wang C., Ren X., Chen Y., Lin P., Zeng L., Shi Z., Li X.J., Shan C.-X. (2021). Ultrabroadband and high-detectivity photodetector based on WS_2_/Ge heterojunction through defect engineering and interface passivation. ACS Nano.

[80] Wang Z., Zhang Z. (2016). Electron beam evaporation deposition. Advanced Nano Deposition Methods.

[81] He L.-J., Li C., Liu X.-Z. (2013). The optical properties of alumina films prepared by electron beam evaporation at oblique incidence. Mater. Lett..

[82] Ripain A.A., Lim Y., Lim C., Zakaria R. (2023). Tailoring of optical and wetting properties of electron beam deposited Ag nanostructure films by oblique angle deposition. J. Opt..

[83] Mazumder J.T., Mayengbam R., Nath A., Sarkar M.B. (2022). Investigation of structural, optical and electrical properties of TiO_2_ thin film-nanowire-based device for photodetector application. Opt. Mater..

[84] Chetri P., Dhar J.C. (2019). Self-powered UV detection using SnO_2_ nanowire arrays with Au Schottky contact. Mater. Sci. Semicond. Process..

[85] Wang X., Liu X., Zou S., Martin P., Bendavid A. (1996). Atomic force microscopy study on topography of films produced by ion-based techniques. J. Appl. Phys..

[86] Jain R.K., Kaur J., Arora S., Kumar A., Chawla A.K., Khanna A. (2019). Effects of oblique angle deposition on structural, electrical and wettability properties of Bi thin films grown by thermal evaporation. Appl. Surf. Sci..

[87] Hassan N., Hashim M., Bououdina M. (2013). One-dimensional ZnO nanostructure growth prepared by thermal evaporation on different substrates: Ultraviolet emission as a function of size and dimensionality. Ceram. Int..

[88] Cansizoglu M.F., Engelken R., Seo H.-W., Karabacak T. (2010). High optical absorption of indium sulfide nanorod arrays formed by glancing angle deposition. ACS Nano.

[89] Yang Z.-P., Ci L., Bur J.A., Lin S.-Y., Ajayan P.M. (2008). Experimental observation of an extremely dark material made by a low-density nanotube array. Nano Lett..

[90] Shahidi M., Ehsani M., Dizaji H.R., Ghazi M. (2020). Photoresponsivity enhancement of SnS porous film. Surf. Interfaces.

[91] Wang F., Zhao H., Liang J., Li T., Luo Y., Lu S., Shi X., Zheng B., Du J., Sun X. (2020). Magnetron sputtering enabled synthesis of nanostructured materials for electrochemical energy storage. J. Mater. Chem. A.

[92] Jain R.K., Gautam Y.K., Dave V., Chawla A.K., Chandra R. (2013). A study on structural, optical and hydrophobic properties of oblique angle sputter deposited HfO_2_ films. Appl. Surf. Sci..

[93] Al-Salman H.S., Abdullah M. (2013). RF sputtering enhanced the morphology and photoluminescence of multi-oriented ZnO nanostructure produced by chemical vapor deposition. J. Alloys Compd..

[94] Yaghoubizadeh P., Eshraghi M.J., Hajati S., Naderi N. (2023). Ultrahigh performance of β-Ga_2_O_3_-based MSM solar-blind photodetectors fabricated via the glancing angle deposition technique. ACS Appl. Electron. Mater..

[95] Ferhati H., Djeffal F., Martin N. (2020). Highly improved responsivity of self-powered UV-Visible photodetector based on TiO_2_/Ag/TiO_2_ multilayer deposited by GLAD technique: Effects of oriented columns and nano-sculptured surface. Appl. Surf. Sci..

[96] Plonczak P., Bieberle-Hütter A., Søgaard M., Ryll T., Martynczuk J., Hendriksen P.V., Gauckler L.J. (2011). Tailoring of La_x_Sr_1-x_CoyFe_1-y_O_3-δ_ nanostructure by pulsed laser deposition. Adv. Funct. Mater..

[97] Soni A., Mulchandani K., Mavani K. (2019). Crystallographically oriented porous ZnO nanostructures with visible-blind photoresponse: Controlling the growth and optical properties. Materialia.

[98] Soni A., Mulchandani K., Mavani K.R. (2020). Effects of substrates on the crystalline growth and UV photosensitivity of glancing angle deposited porous ZnO nanostructures. Sens. Actuators A Phys..

[99] Liu S., Qian Y., Lin Y., Sun L., Zhu Y., Li D. (2024). Multilayer anti-reflective coating with ultra-low refractive index SiO_2_ nanopillars for high efficiency multi-junction GaAs solar cells. Sol. Energy Mater. Sol. Cells.

[100] Khan S.B., Irfan S., Zhang Z. (2024). Fabrication and characterization of MgF_2_ anti-reflective films comprising dual-layer prepared by physical vapor deposition technique for optoelectronic applications. J. Mater. Res. Technol..

[101] Bruynooghe S., Tonova D., Sundermann M., Koch T., Schulz U. (2015). Antireflection coatings combining interference multilayers and a nanoporous MgF_2_ top layer prepared by glancing angle deposition. Surf. Coat. Technol..

[102] De R., Haque S.M., Sikdar M., Sahoo P., Rao K.D. (2021). Fabrication of TiO_2_-based broadband single-layer anti-reflection coating by collimated glancing angle deposition technique. Nanotechnology.

[103] Khan S.B., Wu H., Fei Z., Ning S., Zhang Z. (2017). Antireflective coatings with enhanced adhesion strength. Nanoscale.

[104] Feng C., Zhang W., Wang J., Ma H., Liu S., Yi K., He H., Shao J. (2021). Broadband antireflection film by glancing angle deposition. Opt. Mater..

[105] Guo X., Quan X., Li Z., Li Q., Zhang B., Zhang X., Song C. (2021). Broadband anti-reflection coatings fabricated by precise time-controlled and oblique-angle deposition methods. Coatings.

[106] Liu Y., Zhao Y., Feng Y., Shen J., Liang X., Huang J., Min J., Wang L., Shi W. (2016). The influence of incident angle on physical properties of a novel back contact prepared by oblique angle deposition. Appl. Surf. Sci..

[107] Ferhati H., Djeffal F., Martin N., Benhaya A. (2024). Tunable properties of SnO_x_ sputter-deposited by RGPP and GLAD techniques: A potential candidate for photosensing and all-oxide solar cells. Sol. Energy.

[108] Rana N.K., Debata S., Panda S.K., Singh D.P., Chander N. (2024). Towards water-resistant perovskite solar cells: Electron-beam deposited SiO_2_ layers for highly stable perovskite solar cells. Sol. Energy.

[109] Leem J.W., Yu J.S., Heo J., Park W.-K., Park J.-H., Cho W.J., Kim D.E. (2014). Nanostructured encapsulation coverglasses with wide-angle broadband antireflection and self-cleaning properties for III–V multi-junction solar cell applications. Sol. Energy Mater. Sol. Cells.

[110] Yan X., Poxson D.J., Cho J., Welser R.E., Sood A.K., Kim J.K., Schubert E.F. (2013). Enhanced omnidirectional photovoltaic performance of solar cells using multiple-discrete-layer tailored-and low-refractive index anti-reflection coatings. Adv. Funct. Mater..

[111] Soydan M.C., Ghobadi A., Yildirim D.U., Duman E., Bek A., Erturk V.B., Ozbay E. (2020). Lithography-free random bismuth nanostructures for full solar spectrum harvesting and mid-infrared sensing. Adv. Opt. Mater..

[112] Wu Z., Li P., Zhao J., Xiao T., Hu H., Sun P., Wu Z., Hao J., Sun C., Zhang H. (2021). Low-Temperature-Deposited TiO_2_ Nanopillars for Efficient and Flexible Perovskite Solar Cells. Adv. Mater. Interfaces.

[113] Waita S.M., Aduda B.O., Mwabora J.M., Granqvist C.G., Lindquist S.-E., Niklasson G.A., Hagfeldt A., Boschloo G. (2007). Electron transport and recombination in dye sensitized solar cells fabricated from obliquely sputter deposited and thermally annealed TiO_2_ films. J. Electroanal. Chem..

[114] Cho I., Sim Y.C., Lee K., Cho M., Park J., Kang M., Chang K.S., Jeong C.B., Cho Y.H., Park I. (2023). Nanowatt-Level Photoactivated Gas Sensor Based on Fully-Integrated Visible MicroLED and Plasmonic Nanomaterials. Small.

[115] Lee K., Cho I., Kang M., Jeong J., Choi M., Woo K.Y., Yoon K.-J., Cho Y.-H., Park I. (2022). Ultra-low-power e-nose system based on multi-micro-led-integrated, nanostructured gas sensors and deep learning. ACS Nano.

[116] Kwon Y., Lee K., Kang M., Kim C., Ha J.-H., Han H., Yang S., Yang D., Seo J.H., Park I. (2025). Room-temperature rapid oxygen monitoring system in high humidity hydrogen gas environment towards water electrolysis application. Sens. Actuators B Chem..

[117] Wisitsoorat A., Ahmad M.Z., Yaacob M.H., Horpratum M., Phakaratkul D., Lomas T., Tuantranont A., Wlodarski W. (2013). Optical H_2_ sensing properties of vertically aligned Pd/WO_3_ nanorods thin films deposited via glancing angle rf magnetron sputtering. Sens. Actuators B Chem..

[118] Wolcott A., Smith W.A., Kuykendall T.R., Zhao Y., Zhang J.Z. (2009). Photoelectrochemical study of nanostructured ZnO thin films for hydrogen generation from water splitting. Adv. Funct. Mater..

[119] Wolcott A., Smith W.A., Kuykendall T.R., Zhao Y., Zhang J.Z. (2009). Photoelectrochemical water splitting using dense and aligned TiO_2_ nanorod arrays. Small.

[120] Shi X., Jeong H., Oh S.J., Ma M., Zhang K., Kwon J., Choi I.T., Choi I.Y., Kim H.K., Kim J.K. (2016). Unassisted photoelectrochemical water splitting exceeding 7% solar-to-hydrogen conversion efficiency using photon recycling. Nat. Commun..

[121] Yadav J., Singh J. (2024). Surface plasmonic hot hole driven Ag_2_S/Au/Al_2_O_3_ photocathode for enhanced photoelectrochemical water splitting performance. Renew. Energy.

[122] Yadav J., Bhardwaj L., Singh J. (2024). Magnetic field-augmented photoelectrochemical water splitting in Co_3_O_4_ and NiO nanorod arrays. Mater. Today Energy.

[123] Kawase Y., Higashi T., Obata K., Kishimoto F., Pihosh Y., Domen K., Takanabe K. (2024). Simple Immersing Method of Nanocoating on Uneven Surfaces Applicable to Highly Durable Ta_3_N_5_ Nanorod Photoelectrode for Water Splitting. Chem. Mat..

[124] Pihosh Y., Turkevych I., Mawatari K., Uemura J., Kazoe Y., Kosar S., Makita K., Sugaya T., Matsui T., Fujita D. (2015). Photocatalytic generation of hydrogen by core-shell WO_3_/BiVO_4_ nanorods with ultimate water splitting efficiency. Sci. Rep..

[125] Wu S., Ou K., Zhang W., Ni Y., Xia Y., Wang H. (2024). TiO_2_ nanorod arrays/Ti_3_C_2_T_x_ MXene nanosheet composites with efficient photocatalytic activity. Nanotechnology.

[126] Swathi S., Makkaramkott A., Subramanian A. (2023). Tin oxide nanorod array-based photonic memristors with multilevel resistance states driven by optoelectronic stimuli. ACS Appl. Mater. Interfaces.

[127] Jeon Y., Lee G., Kim Y.J., Jang B.C., Yoo H. (2024). Dual Synapses and Security Devices from Ternary C_60_-Pentacene-TiO_2-x_ Nanorods Heterostructures. Adv. Funct. Mater..

[128] Priyadarshini B.G., Sharma A.K. (2016). Design of multi-layer anti-reflection coating for terrestrial solar panel glass. Bull. Mat. Sci..

[129] Hiller J.A., Mendelsohn J.D., Rubner M.F. (2002). Reversibly erasable nanoporous anti-reflection coatings from polyelectrolyte multilayers. Nat. Mater..

[130] Lee G., Kim Y.E., Kim H., Lee H.K., Park J.Y., Oh S., Yoo H. (2025). Organic Synaptic Transistors and Printed Circuit Board Defect Inspection with Photonic Stimulation: A Novel Approach Using Oblique Angle Deposition. Small.

[131] Shi J., Zhang J., Yang L., Qu M., Qi D.C., Zhang K.H. (2021). Wide bandgap oxide semiconductors: From materials physics to optoelectronic devices. Adv. Mater..

[132] Pei J., Wu X., Liu W.-J., Zhang D.W., Ding S.-J. (2022). Photoelectric logic and in situ memory transistors with stepped floating gates of perovskite quantum dots. ACS Nano.

[133] Fang X., MacDonald K.F., Zheludev N.I. (2015). Controlling light with light using coherent metadevices: All-optical transistor, summator and invertor. Light Sci. Appl..

[134] Cheng J., Wang C., Zou X., Liao L. (2019). Recent advances in optoelectronic devices based on 2D materials and their heterostructures. Adv. Opt. Mater..

